# Accuracy of day-to-day patient positioning in ocular proton therapy with a dedicated beamline

**DOI:** 10.1371/journal.pone.0333294

**Published:** 2025-09-29

**Authors:** Martijn Hol, Kees H. Spruijt, Myra F. Rodrigues, Yvonne L. B. Klaver, Jasper Kouwenberg, Pauline A. C. Bakker, Gregorius P. M. Luyten, Emine Kiliç, Jan-Willem M. Beenakker, Eleftheria Astreinidou, Coen R. N. Rasch

**Affiliations:** 1 Department of Radiation Oncology, Leiden University Medical Center, Leiden, The Netherlands; 2 HollandPTC, Delft, the Netherlands; 3 Department of Ophthalmology, Leiden University Medical Center, Leiden, The Netherlands; 4 Department of Ophthalmology, Erasmus Medical Center, Rotterdam, The Netherlands; 5 Department of Clinical Genetics, Erasmus Medical Center, Rotterdam, The Netherlands; 6 Department of Radiology, Leiden University Medical Center, Leiden, The Netherlands; Northwestern University Feinberg School of Medicine, UNITED STATES OF AMERICA

## Abstract

The purpose of this study was to determine the residual patient positioning error of ocular proton treatment based on daily setup X-ray images and tantalum clips. The residual day-to-day patient positioning accuracy in a cohort of 60 patients and 240 fractions was determined in two steps. The 3D tantalum clips’ positions in the orthogonal X-ray images just prior to aperture placement were registered with 3D clips’ positions derived from the reference data from treatment planning. The aperture, placed before irradiation, reduced the field of view in axial images blocking clips partially or completely in most cases. The clips’ positions after aperture placement in the lateral images were compared with the reference clips’ positions. Partially visible clips in the axial image were registered to the clips in the axial image prior to aperture placement. The day-to-day deviations to aperture placement were corrected with the registration results for translations only. The residual day-to-day patient position variations just prior to irradiation were −0.06 ± 0.27 mm (mean ± 1SD) for the left-right direction, −0.01 ± 0.23 mm for the anterior-posterior direction and −0.01 ± 0.19 mm for the superior-inferior direction. This data was further investigated by differentiating for treated eye, gazing eye and the use of eyelid retractor. This yielded no significant differences. The residual patient positioning errors are small for patients treated at the dedicated eyeline, indicating that the patient positioning workflow using tantalum clips is highly accurate. The reported errors can be used for ocular proton margin calculations and for the evaluation of a clipless ocular proton therapy workflow.

## Introduction

Uveal Melanoma is the most common primary malignant intraocular lesion comprising of 242 out of 265 ocular malignancies in 2019 (incidence 1.4/100.000) in The Netherlands [1]. Local treatment options include enucleation, brachytherapy and external beam radiation therapy (photon or charged particle therapy). External beam therapy is used for patients with a tumor larger than 7 mm in thickness and/or larger than 16 mm in diameter and/or close (<2mm) to the optic disc [2]. Proton therapy makes use of the steep penumbra and dose falloff enabling sparing of the critical structures such as optic disc and optic nerve, macula, ciliary body and lens and has yielded good results with 5-years local tumor control up to 95% and an eye retention rate of 90% [3–6]. In the beginning of 2020, the first ocular patient was treated at the ocular Proton beamline (Varian Medical Systems Particle Therapy GmbH & Co. KG, Troisdorf, Germany) at HollandPTC (Delft, The Netherlands).

### HollandPTC Eyeline and workflow

The HollandPTC eyeline uses a cyclotron for proton beam generation and a dedicated nozzle as described by Fleury et al [7]. The treatment room further consists of a robotic chair including a patient mask holder, two X-ray sources and two flat imaging panels for patient positioning verification, and a close-up video system to monitor the treated eye. The proton eyeline workflow [8–10] consist of six phases: pretreatment imaging, clip placement, simulation run, treatment planning, dry run and radiation treatment session.

To facilitate target definition and patient alignment, three to four tantalum clips (Altomed Limited, Boldon, England, modelnumber: A7198S) surrounding the transilluminated base of the tumor are sutured onto the sclera during surgery [11]. The tantalum clips have a diameter of 2.5 mm and thickness of 0.17 mm. During surgery the ophthalmologist surgeon will measure clip-to-clip and tumor-to-clip distances. The placement of the clips is performed at Leiden University Medical Center or at Erasmus Medical Center, two weeks prior to the proton therapy treatment.

In the simulation phase, the patient is positioned in a robotic chair in an upright position. The affected eye is anesthetized with eye drops and kept open with an eyelid retractor (Barraquer eye speculum (40 mm), Vision Medical Company) or by adhesive strips (Hartman Omnitape). An individualized thermoplastic mask (Qfix, Aquaplast BoS RT1882, Avondale, PA, USA) including bite-block (Orbis A silicone putty), attached to a head frame and tightened with a strap at the back of the patient’s head, is used for securing the patient positioning. The patient will look at a light emitting diode (LED) to establish the gazing angle of the affected eye. Sets of orthogonal X-ray images are acquired with different gazing angles. For each patient, the optimal gazing angle is selected during treatment planning, based on the dose to the organs at risk. Maximum gazing angles are determined in a simulation session before treatment planning, in which the patients’ eye movement range is determined, and gazing stability of these positions is verified. Gazing towards the LED is done by default with the affected eye. Only when not stable enough, the healthy contralateral eye will be used.

The X-ray imaging sets together with the measurements of the surgeon, fundus imaging and MRI based measurements [11,12] are used in the Eclipse Ocular Proton Planning (Version 13.5.01) as input for the 3D geometric model of the eye. Based on the dose to the organs at risk, the optimal gazing is determined, since one gazing angle will be used during the treatment. Each patient at HollandPTC will receive a total prescribed relative biological effectiveness (RBE, RBE factor of 1.1) equivalent dose of 60 Gy(RBE) in four fractions.

**Fig 1:**
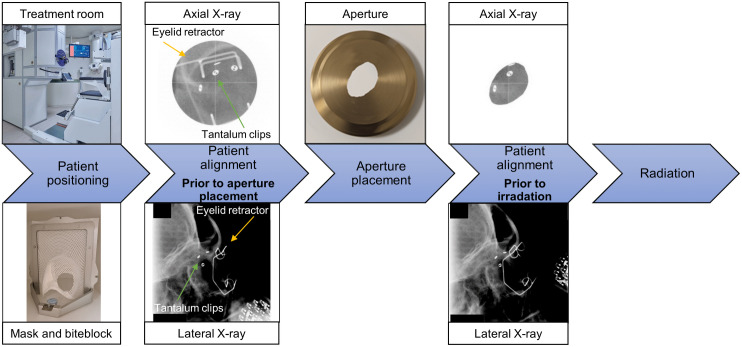
Ocular proton patient positioning worklow. Ocular patient positioning workflow at HollandPTC. The patient sits on the robotic chair and a mask with bite-block is applied. X-ray images are made for patient alignment. The aperture is attached to the nozzle, the final patient alignment is performed with X-ray imaging. The field of view of the axial X-ray image is potentially reduced after the aperture placement.

The treatment sessions take place on four consecutive days. The patient stares towards the gazing LED, X-ray images are acquired and compared to the reference clip structures provided by the treatment planning system. Fig 1 shows the patient positioning workflow. The registration is done manually by the radiotherapy technicians: deviations are corrected by the robotic chair for translation only, until the patient is within 0.2 mm deviation per direction. However, primarily due to rotational deviations, this level of precision is attainable only in a select subset of patients. In such cases, alignment is optimized to minimize overall error, with increased tolerance permitted in the anterior-posterior direction. This is justified by the fact that the proton beam enters along this axis, making it less susceptible to minor inaccuracies.

After the initial patient alignment, the robotic chair is retracted, the aperture is inserted and the robotic chair is moved back to its position after the initial alignment. As the aperture reduces the field of view in the axial X-ray image, in most of the cases only a subset of the clips is visible for this final positioning. X-ray images are used to validate the patient position using the still visible clips and, if required, the patient position is corrected by the robotic chair until the patient is within 0.2 mm per direction. After the patient alignment, the eye position is monitored by live video and the irradiation is started. During the irradiation, which takes approximately 60 sec, technicians will visually monitor the pupil location, and stop the irradiation and reposition the patient if required*.*

### Patient positioning errors

The safety margin used at HollandPTC and applied to the gross tumor volume (GTV), which is defined by a radiation oncologist, is the commonly used 2.5 mm [8–10]. This safety margin addresses uncertainties like tumor delineation, three-dimensional (3D) eye modeling, X-ray vs proton co-incidence, absolute range accuracy, the aperture manufacturing accuracy, penumbra accuracy (50%−90% penumbra), intra-fractional eye movement and patient positioning. The patient positioning on a dedicated beamline is performed using tantalum clips, which surround the transilluminated base of the tumor and are sutured onto the sclera during surgery, and orthogonal X-ray imaging [4,9]. In current literature (based on PubMed and Google scholar searches), the positioning accuracy for ocular proton therapy has only been reported for a relative small cohort of 20 patients on a non-dedicated proton eyeline [13]. The aim of this study is to determine the residual patient positioning accuracy during the ocular proton treatment on a dedicated proton eyeline to facilitate safety margin calculations, such as described by Wulff et al. [14].

## Methods and materials

This retrospective study includes 60 consecutive patients treated for uveal melanoma, 60Gy(RBE) in 4 fractions. The ethics committee (METC-LDD) received the research protocol (P18.053) and waived the need to access the protocol. However, all patients signed consent to use their data for research purposes (this includes the specific study of the research in this paper). The medical records were anonymized and accessed at 14 August 2023 and the authors had no access to information that could identify individual participants. The average age of the cohort was 67.5 ± 11.9 years (mean ± 1SD). The average apex height and the maximum base diameter were 7.8 ± 3.5 mm and 18.1 ± 3.9 mm, respectively.

To determine the daily variation in patient positioning for the selected cohort, the clips’ positions (typically four clips per patient), retrieved from X-ray imaging, were compared to the digitally reconstructed radiographs (DRR). The latter given by the treatment planning system. In total 240 fractions were retrospectively analysed. DRR data is based on X-ray images acquired on the same imaging system as used during treatments, in contrast to current conventional radiotherapy where typically 3D imaging such as Computed Tomography (CT) or Magnetic Resonance Imaging (MRI) is used. Analyses were performed with Python version 3.9 and Skimage Image Registration library (scikit-image version 0.19.3). The validation of the registration methods can be found in S1 Appendix: Registration validation.

### Clip-to-clip distances

By determining the clip-to-clip distances in the X-ray images and comparing them to the reference clip-to-clip distances obtained for the DRR data, the stability of the used method of obtaining the clips’ positions from the reference DRR and the X-ray images was investigated. The clip-to-clip distances were calculated between the clips’ positions resulting from constructed 3D clip position from the two-dimensional (2D) reference DRR data and the X-ray images. A comparison was made between the distances retrieved from the reference DRR and the distances found in the X-ray images for each patient and each fraction.

### Clips’ positions determination from DRR (reference)

**Fig 2:**
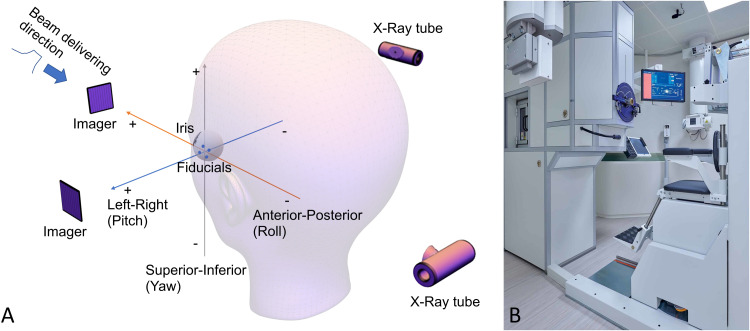
Patients’ orientation to HollandPTC treatment rooms and beam alignment. (A) Patient positioning in relation to the imaging system and proton beam direction. (B) Eyeline treatment room at HollandPTC.

The reference clips’ positions were extracted from the axial and lateral reference files with an in-house developed script that calculated the center position per clip using the center of mass. The 2D center positions in the axial and lateral direction per clip were combined to a 3D coordinate, where an average was calculated to mitigate for variations in the superior-inferior values of the axial and lateral image. Fig 2 shows the orientation.

### Clips’ positions determination from X-ray images

For each fraction, the clips’ positions in the two orthogonal X-ray images (80-100 kVp, 25–56 mAs), acquired just prior to aperture placement, were correlated with the clips’ reference positions. The lateral and axial flat panel imagers (PaxScan 1313DX, Varian Medical Systems Particle Therapy GmbH & Co. KG, Troisdorf, Germany) have a resolution of 1024x1024 and a pixel size of respectively 0.082 and 0.101 mm/pixel at isocenter. The source-to-imager distance for the lateral X-ray configuration is 2779 millimeter, and for axial configuration 3013 millimeter and source-to-axis distances are respectively 1805 and 2400 millimeter.

The center position of the clips in the two radiographs were localized manually using ImageJ (Rasband, W.S., ImageJ, U. S. National Institutes of Health, Bethesda, Maryland, USA). 3D coordinates were created from the 2D clips’ positions in the lateral and axial image. The registration of the reference clips’ positions and determination of clips’ positions from the X-ray images were done with a least-squares algorithm.

Determining the residual patient positioning prior to irradiation presents challenges as the patient-specific aperture obscures one or more clips for most patients (Fig 1). Therefore both positioning accuracy prior and after aperture placement are assessed separately. Before aperture placement, all clips are visible on the X-ray images and therefore both translational and rotational positioning errors will be obtained. After aperture placement, for the clips that were not obscured by the aperture, their 3D location was obtained similarly as before aperture placement. For the clips that were only partially visible in the axial images, a masked registration with the pre-aperture placement X-ray images was performed. This registration was performed for translations only, as the rotations could not accurately be derived due to the (partial) visibility of the clips. The deviations between clips’ positions prior to aperture placement in the left-right direction were corrected with the registration results.

### Statistics

For each patient and for each direction, the mean and standard deviation of the X-ray clips’ positions in comparison to the clips’ positions retrieved from the treatment planning system were determined. This data was used to calculate the systematic error of the entire patient population and the population random error [15]. The systematic error is given by,


$$\Sigma \;=\;{SD}_{p}(S_{p})$$ (1)

Where SD_p_ is the standard deviation of the mean systemic error per patient S_p_.

The population random error was calculated by


$$\sigma =\sqrt{\left\langle \sigma_{p}^{\text{2}}\right\rangle }$$ (2)

Where σ_p_ is the variance per patient.

The systematic contribution can be divided into several components, namely alignment of the X-ray system with respect to the proton beam, patient setup error, treatment planning model size and shape, range difference in regards to the treatment planning system, stopping power used for the eye, and accuracy of the aperture manufacturing. The systematic patient setup error consists of the alignment of the X-ray system, shifting accuracy of the chair, and incorrect calibration of the system. If the calibrations of the system are good and the overall system is functioning well, one would expect that the average error for a large cohort is around zero. To ensure that systematic errors are minimized, quality control measurements are performed (including the X-ray and proton beam coincidence and aperture manufacturing) on the different items of the whole chain. The random errors are due to patient eye positioning and Bragg peak accuracy. Based on the different errors margins can be calculated as shown by Wulff et al [14].

The clips positioning deviations data were further analysed by differentiating for the treated eye, the gazing eye and the use of an eyelid retractor. Since these parameters show normally distributed behavior, an independent two-tailed t-tests was performed comparing the means of the different groups. Furthermore, an analysis of variance (ANOVA) was conducted to evaluate a potential correlation between the gazing angle (polar angle and azimuthal angle) and the absolute deviation. Statistical tests were performed in ScyPi version 1.10.1 and a significance level of p < 0.05 was applied.

## Results

### Clip-to-clip distances

**Fig 3:**
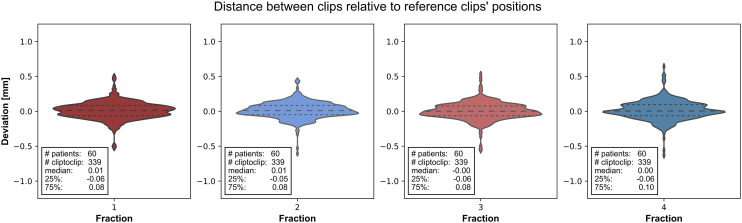
Clip-to-clip distance deviations. Violin plots showing the clip-to-clip deviations of the treatment fractions relative to the reference clips’ positions. Per fraction the median, the 25th and the 75th percentile are given of the distribution.

For two patients in the cohort of 60 patients only three clips were visible in the X-ray imaging set, where for five patients only three of the four clips were clearly visible in the axial X-ray image. In total, 339 clip-to-clip distances per fraction extracted from the X-ray image data before aperture placement were analysed in reference to the clip-to-clip positions in the reference DRR. The mean deviation for each fraction was 0.1 mm or less with a standard deviation less than 0.1 mm. This indicates that the used method for establishing the clips’ positions from the reference DRR and the X-ray images was accurate. Fig 3 shows the distribution of clip-to-clip deviation for each fraction in a violin plot.

### Patient positioning

**Fig 4:**
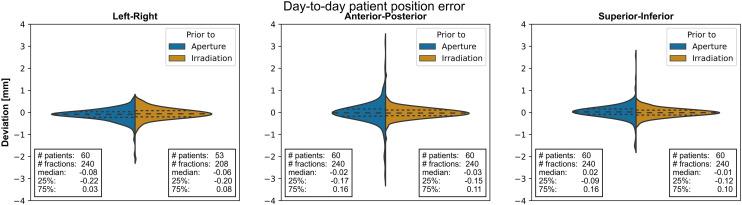
Patient positioning accuracy distribution. Violin plots indicating translational patient positioning deviations prior to aperture placement and treatment. For each direction the median, the 25th and the 75th percentile of the distribution are given.

The translation and rotation axes are shown in Fig 2. Table 1 shows the results based on the imaging just prior to the aperture placement and prior to irradiation. For the patient positioning prior to irradiation, the anterior-posterior and superior-inferior were determined on the lateral X-ray images of 240 fraction. In the left-right direction the data was calculated on a dataset of 53 patients and 208 fractions (in fractions of different patients, clips in the axial image after the aperture placement were not visible). The number of clips available for registration per fraction were: one clip for 57 fractions, two clips for 111 fractions, three clips for 16 fractions and all clips in 24 fractions. The violin plot, showed in Fig 4, displays the distribution of the translation deviations among the fractions for the data prior to aperture placement and prior to irradiation.

**Table 1:** Patient positing accuracy.

	Patient positioning prior to aperture placement					
	Left-Right [mm]	Anterior-Posterior [mm]	Superior-Inferior [mm]	Pitch [degree]	Roll [degree]	Yaw [degree]
Mean deviation	−0.11	−0.03	0.03	−0.07	0.30	−0.23
Standard deviation	0.28	0.59	0.36	1.93	2.10	2.43
Variance random error	0.24	0.44	0.31	1.09	0.73	0.91
Variance systematic error	0.19	0.47	0.24	1.69	2.01	2.31
	**Patient positioning prior to irradiation**					
	**Left-Right [mm]**	**Anterior-Posterior [mm]**	**Superior-Inferior [mm]**			
Mean deviation	−0.06	−0.02	−0.01			
Standard deviation	0.28	0.25	0.19			
Variance random error	0.25	0.24	0.14			
Variance systematic error	0.19	0.13	0.15			

Day-to-day patient positioning statistics prior to aperture placement based on 240 fractions and prior to irradiation based on 208 fractions for the left-right direction and on 240 fractions for the anterior-posterior and superior-inferior directions. Positive translations correspond with translation in the Left, Anterior, Superior direction. Rotational movements are considered positive when counterclockwise and negative when clockwise.

**Fig 5:**
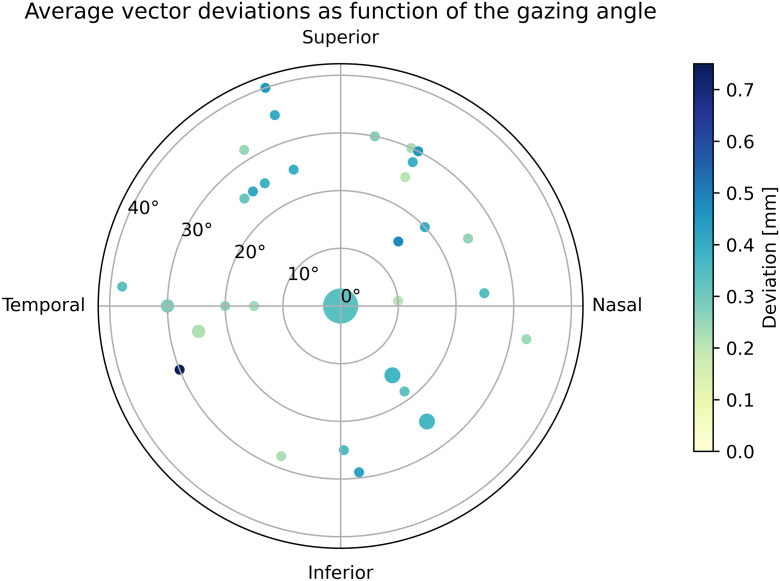
Vector deviations as function of the gazing angle (polar and azimuthal angle). The polar plot showing no significant (p > 0.2) correlation between both polar and azimuthal angle and deviation. The size of the marker gives an indication of the number of data points.

The patient positioning accuracy prior to irradiation was further investigated by the following parameters: treated eye (right versus left), gazing eye (affected versus contralateral) and the use of an eyelid retractor (yes versus no). For the treated eye (right vs left, Superior-Inferior direction), the p-value (0.004) indicated a significant difference, although the found difference, right eye −0.04 ± 0.19 mm versus left eye 0.03 ± 0.18 mm, is not of clinical relevance. The p-values of the other t-tests were higher than the significance level of 0.05. No significant (p > 0.2) correlation between the gazing angle and absolute deviations of the patient positioning was found, as can be seen in Fig 5.

## Discussion

In this study, the residual patient positioning error was established in the proton ocular workflow using tantalum clips for a dedicated beamline. The determination of the day-to-day patient positioning accuracy just prior to irradiation raises challenges. The aperture limits the field of view of the axial X-ray images, and therefore only a subset of the clips is partly visible in most of these axial images. In these cases, the available clips were registered for translation only to the clips in the axial X-ray image just prior to the aperture placement. The resulting registration was applied to the match of the 3D clips’ positions in the X-ray images set prior to the aperture placement and the 3D clips positions of the reference of the left-right direction only. Due to missing clips in the axial image, no rotational deviation was calculated and the reliability of the translational registration was mitigated. The residual errors for other directions were retrieved from the comparison between the clips’ positions in the lateral X-ray image and the clips’ positions in the lateral reference DRR. Another limitation of this study is the absence a validation of the used method against clinical derived registration shifts, as the results of the clinically performed manual registrations were not available.

The mean residual errors of the patient population, thus prior to irradiation, were less than 0.1 mm in each direction indicating that the current HollandPTC patient positioning workflow is highly accurate. The variations in the rotations in this study were −0.07 ± 1.93 degree in the roll direction, 0.30 ± 2.10 degree in the pitch direction and −0.23 ± 2.43 degree in the yaw directions. The yaw rotation can be linked to the azimuthal angle, whereas pitch rotation is related to the polar angle. The rotational deviations were calculated in the center of the clips surrounding the tumor, close to the isocenter of the treatment plan. This is not the center of the eye, which means that there is not a direct link between the gazing angle and the rotational deviations presented in this study. The data was based on the three-dimensional position of the clips in the reference data and the X-ray images and are thus only calculated for the X-ray imaging sets acquired just before the aperture placement.

Considering the variations in rotations, studies by Muller et al [16] and Dieckmann et al. [17] showed that high deviations in ocular rotation do not automatically lead to significant displacement of the target. The threshold used for registration is 0.2 mm. As the clips are located closely to the isocenter, deviations associated with rotational mismatches are minimal and therefore no additional threshold is required.Via et al. [13] reported the patient positioning accuracy at Centro Nazionale di Adroterapia Oncologica (CNAO, Pavia, Italy) in a smaller group of 20 patients treated at a non-dedicated beamline instead of a dedicated beamline in this study. In the study of Via et al., a different analysis was performed: patient positioning errors were indirectly determined. This model was validated with a median 3D reconstruction error of 0.41 mm (IQR: 0.24 mm). For the 20 patients the median residual registration error after patient positioning found was −0.17 mm (IQR: 0.30 mm) in lateral direction, 0.03 mm (IQR: 0.19 mm) in longitudinal direction and −0.11 mm (IQR: 0.42 mm) in vertical direction. These results are similar to the data presented in this publication however with a median error less concentrated around zero.

The observed random and systematic errors align closely with the values proposed by Wulff et al. [14]. Although these small values might suggest the potential for a reduction of the 2.5 mm margin, such decisions must account for all sources of uncertainty, including those related to target definition, which are likely to be the dominant contributors to the overall margin.

The low contrast characteristics of the X-ray imaging is poor, therefore the tumor is not visible on the X-ray images. The tantalum clips act as a surrogate for the tumor location and are used during treatment planning and patient positioning. Clipless treatment will elevate the comfort of the patient, but requires developments in different areas of the proton ocular treatment. Currently developments are seen in the use of 3D imaging, such as MRI, in treatment planning for eye modeling and delineation [11,18–20]. Furthermore, an alternative procedure is needed for patient alignment during clipless treatment. The application of an optical eye tracking system to validate the eye gazing position during treatment is investigated at both the Paul Scherrer Institute (Villigen, Swiss) and CNAO [13,21,22]. The patient positioning accuracy for an ocular proton workflow using tantalum clips with a dedicated beamline, presented in this paper, can be used as a benchmark for the evaluation of a clipless treatment.

In this study the accuracy of the day-to-day patient positioning using tantalum clips was evaluated for a cohort of 60 consecutive ocular patients receiving proton therapy at HollandPTC on a dedicated eyeline. The day-to-day patient positioning based on the X-ray imaging data just prior to the irradiation were −0.06 ± 0.27 mm (mean ± 1SD) for the left-right direction, −0.01 ± 0.23 mm for the anterior-posterior direction and −0.01 ± 0.19 mm for the superior-inferior direction. The residual translation mean errors of the tantalum clips alignment was accurate (<0.1 mm) without significant systematic errors, meaning that patient positioning is performed with a high level of accuracy.

## Supporting information

S1 Appendix Registration validation. (PDF)
