# Supplementary material for: Accuracy of day-to-day patient positioning in ocular proton therapy with a dedicated beamline
Source: PLoS One. 2025 Sep 29;20(9):e0333294. doi: 10.1371/journal.pone.0333294 (PMC12478952; doi:10.1371/journal.pone.0333294)
Supplement: S1 Appendix — (PDF) [file pone.0333294.s001.pdf]

S1 Appendix

Registration validation

The validation of the registration workflow of the clips was performed separately on a conventional linear accelerator Elekta Synergy (Elekta, Crawley, UK) with the Ball Cube I (Accuray Incorporated, Sunnyvale, CA) phantom containing 5 golden markers, Protura 6 degrees-of-freedom couch (CIVCO Medical Solutions, Kalona, IA, USA) and X-ray planarviews by Elekta XVI system (Elekta, Crawley, UK). The phantom was positioned at 14 combinations of translations and rotations for which a set of X-ray images was acquired. The marker locations and subsequent registration to the first position, were performed similarly to the described workflow for the clip detection and registration, and compared to the set rotation and translation of the Protura couch. The results of the registration was then compared to the planned movement. Table 1 shows the deviation of the registration in comparison to the known movement.

Table 1: Validation registration results

	Planned Translation and Rotation shift						Least square registration deviation					
Set #	X [mm]	Y [mm]	Z [mm]	Xr [degree]	Yz [degree]	Zr [degree]	X [mm]	Y [mm]	Z [mm]	Xr [degree]	Yr [degree]	Zr [degree]
1	0,0	0,0	0,0	2,0	0,0	0,0	-0,1	-0,1	-0,3	0,0	0,0	0,1
2	0,0	0,0	0,0	0,0	2,0	0,0	-0,1	-0,1	-0,1	0,1	-0,2	0,0
3	0,0	0,0	0,0	0,1	0,0	2,0	-0,3	-0,1	0,0	0,1	-0,1	0,1
4	0,0	5,0	10,0	-1,0	0,0	0,0	0,0	-0,1	-0,1	0,2	0,0	-0,1
5	-10,0	-5,0	0,0	0,0	0,0	-1,0	-0,2	0,0	0,0	-0,2	-0,3	0,2
6	5,0	0,0	-10,0	0,0	-1,5	0,0	0,1	0,0	-0,2	-0,1	0,1	0,2
7	5,0	0,0	-10,0	2,0	-2,5	0,0	0,0	0,0	-0,4	-0,1	0,0	0,3
8	0,0	0,0	0,0	0,0	-1,0	-2,0	0,1	0,0	-0,2	0,0	-0,1	0,2
9	5,0	-7,5	10,0	2,0	-1,0	0,0	-0,2	0,0	-0,3	-0,1	0,1	0,1
10	-10,0	5,0	-7,5	0,0	-1,0	-2,0	0,1	0,0	0,0	0,1	-0,4	0,1
11	7,5	-10,0	5,0	-1,5	2,0	1,0	-0,2	0,0	0,2	-0,1	0,0	0,3
12	-0,3	0,2	-0,3	-1,5	2,0	1,0	-0,2	-0,1	0,1	0,1	-0,2	0,1
13	0,5	-1,0	0,5	2,1	-1,5	2,0	-0,2	-0,1	-0,3	0,0	-0,1	0,0
						**Mean**	**-0,1**	**0,0**	**-0,1**	**0,0**	**-0,1**	**0,1**
						**Stdev**	**0,1**	**0,1**	**0,2**	**0,1**	**0,2**	**0,1**

The found deviations were small especially when the pixelsize of 0.26mm was taken into consideration.

Besides the validation with the Protura 6 degrees-of-freedom couch (CIVCO) and the ballcube, the graphical output of the registration also gave an insight into the registration accuracy. For each patient and fraction the registration results were shown in 3D and per projections (0 and 90 degrees). The fig 1 and 2 show respectively the 3D and projection results of one of the fraction of a patient.

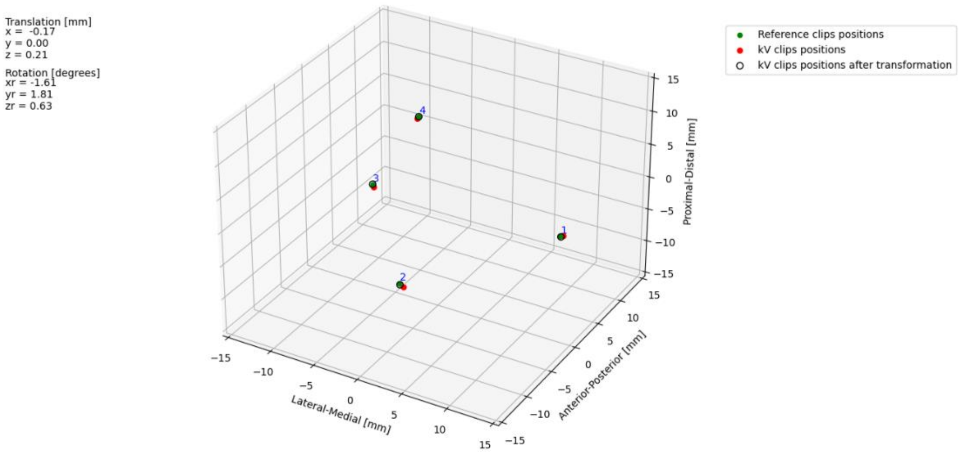

Fig 1: 3D registration results of a fraction of a patient

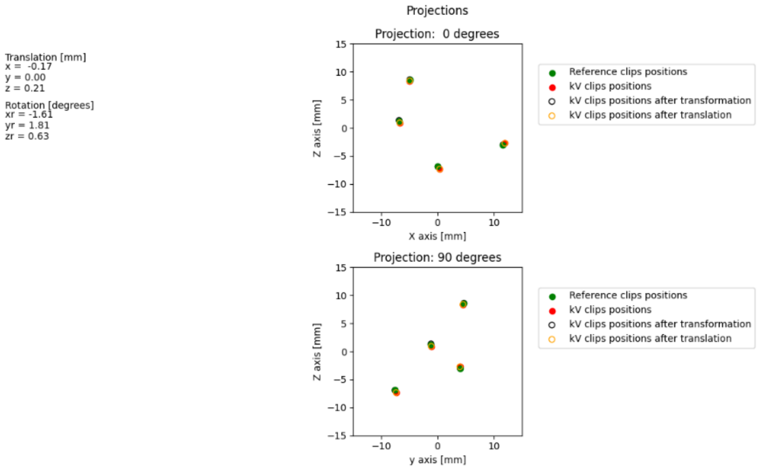

Fig 2: Projections registration results of a fraction of a patient

The registration results for the axial images before and after the aperture placement were validated visually by overlaying the two images before and after registration. As shown in fig 3 a clear deviation is seen in purple and green when the registration is not yet performed.

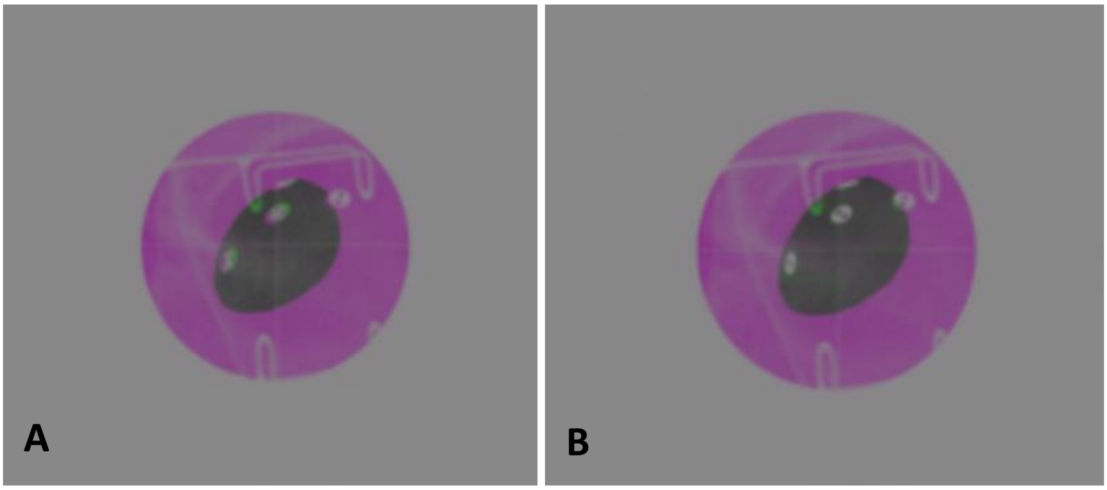

Fig 3: Registration results (A: before registration, B: after registration) on the clips of Axial X-ray images before and after aperture placement.
